# Unusual Case of Cervical Tissue Penetration by a Wool‐Like Foreign Body Inserted for Male Child Conception

**DOI:** 10.1155/crog/8965121

**Published:** 2026-06-07

**Authors:** Alireza Babajani, Sayna Abbaszadeh, Fatemeh Ayatollahi, Mahnaz Faramarzi Kheneshti

**Affiliations:** ^1^ Department of Anesthesiology, School of Allied Medical Sciences, Alborz University of Medical Sciences, Karaj, Iran, abzums.ac.ir; ^2^ Department of Obstetrics and Gynecology, School of Medicine, Khoy University of Medical Sciences, Khoy, Iran; ^3^ Department of Obstetrics and Gynecology, Khoy University of Medical Science, Khoy, Iran; ^4^ BSc Operation Room, Shahriyar Social Security Hospital, Social Security Organization, Tehran, Iran

**Keywords:** case report, cervical inflammation, conception, vaginal discharge, vaginal foreign body

## Abstract

Retention of a foreign body in the vagina can occur intentionally or unintentionally. This condition can lead to a variety of clinical symptoms. We present a case involving a 39‐year‐old multiparous woman with an obstetric history comprising four pregnancies, three term vaginal deliveries and one spontaneous miscarriage, who was hospitalized on January 17, 2024, for acute management of profuse vaginal discharge and pyrexia secondary to the intravaginal retention of a wool‐like foreign body. Clinical examination revealed a woolen foreign body in the cervix, inserted 4 years earlier as a traditional method to influence fetal sex. Pelvic ultrasound showed a spongy mass (50 × 30 mm) in the vaginal canal. Vaginoscopy and hysteroscopy performed under general anesthesia facilitated the identification and extraction of a 50 × 50 mm malodorous, wool‐like foreign body embedded 5 mm within the cervical stroma. Biopsies confirmed a foreign body reaction with acute and chronic inflammation, reactive epithelial changes, and keratin‐like fibers infiltrating the cervical stroma. Intraoperative assessment revealed no extension to the uterine cavity or urinary bladder. The patient was administered a 72‐h course of empirical antibiotic therapy, achieved complete clinical resolution, and was subsequently discharged with recommendations for ambulatory follow‐up. The retention of a foreign body within the vaginal vault may engender a spectrum of clinical manifestations and sequelae, contingent upon the nature of the foreign material and the duration of its persistence. Prolonged indwelling of such objects can precipitate histopathologic alterations in contiguous tissues, potentially mandating surgical extirpation and serial surveillance evaluations.

## 1. Introduction

The presence of a foreign body in the vagina can occur intentionally or unintentionally. This condition can lead to a variety of clinical symptoms, including infectious and malodorous vaginal discharge. If left untreated, such cases may progress to complications, including chronic inflammation, epithelial alterations, and, in rare instances, malignant transformation [1].

This report describes the prolonged intravaginal retention of a wool‐like object, a phenomenon infrequently documented in the literature. The objective of this study is to underscore the clinical implications of long‐standing vaginal foreign bodies, outline appropriate diagnostic and therapeutic approaches, and enhance awareness between both the general public and healthcare practitioners. It is noteworthy that the present report has been prepared in accordance with the care checklist.

## 2. Case Presentation

A 39‐yea‐ old woman, gravida 4 para 3, with a history of three‐term vaginal deliveries and one spontaneous miscarriage that occurred at home 4 years earlier, was admitted on January 17, 2024, with complaints of severe vaginal discharge and fever. On admission, her vital signs indicated tachycardia at 130 beats per minute; blood pressure of 115/70 mmHg, and a body temperature of 38.7°C. Laboratory evaluation demonstrated a semiquantitatively elevated C‐reactive protein (CRP) level graded as 1+, an erythrocyte sedimentation rate (ESR) of 13 mm/h, and a leukocyte count of 7.6 × 10^3^/*μ*L. Initial pelvic examination revealed the presence of a woolen foreign body lodged within the cervix. The patient reported intentionally inserting this material into the cervical canal 4 years prior as part of a traditional ethnomedical practice believed to influence fetal sex determination. Notably, she reported no conception during the intervening years and described her menstrual cycles as consistently regular, without abnormalities.

**Figure 1:**
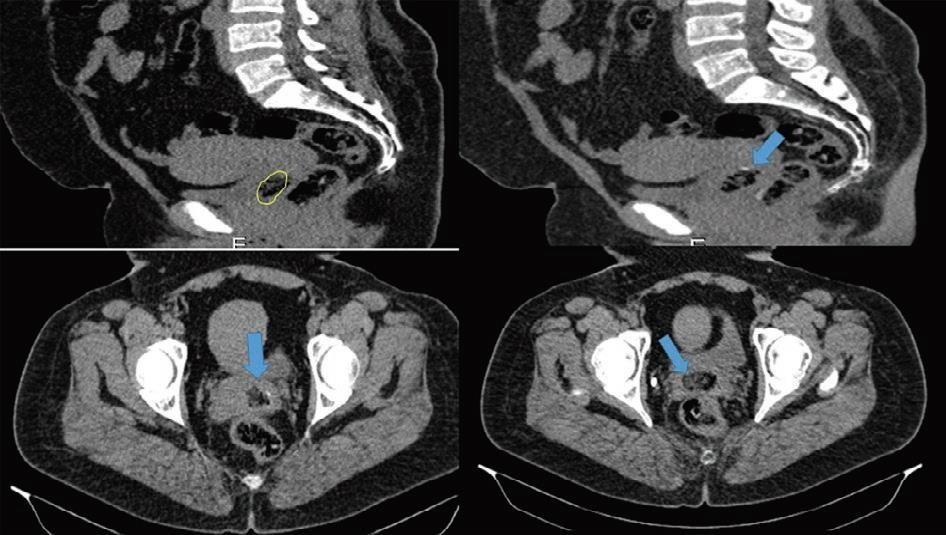
Computed tomography (CT) images reconstructed in sagittal and axial planes.

In accordance with clinical guidelines, pelvic ultrasonography and abdominopelvic‐computed tomography (CT) were performed. Imaging studies revealed no pathological abnormalities in the solid abdominal organs, no free intraperitoneal fluid, and no hydronephrosis. However, a spongy hypoechoic lesion measuring 50 × 30 mm with a central calcified core was visualized in the upper vaginal canal, corroborating the findings of the clinical examination. Representative CT images were presented in Figure 1.

**Figure 2:**
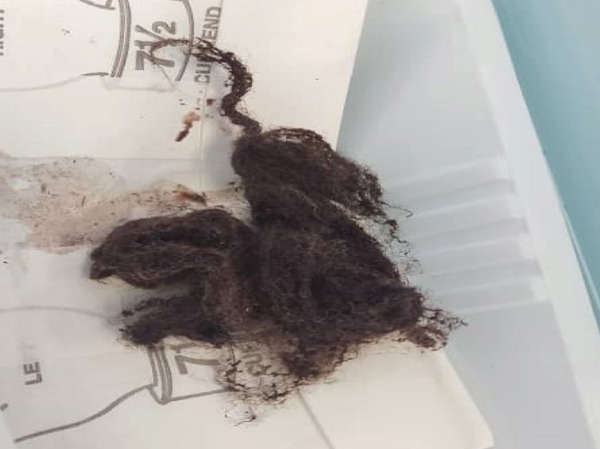
Wool‐like foreign body protruding from the cervical opening.

**Figure 3:**
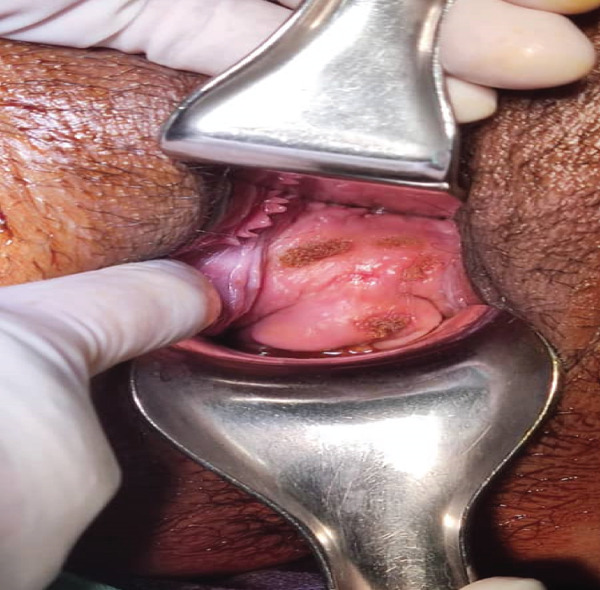
Penetration of a foreign body into the anterior and posterior cervical tissues.

The patient was transferred to the operating theater and placed under general anesthesia for combined vaginoscopy and hysteroscopy. Upon insertion of the speculum, a 50 × 50 mm malodorous, woolen‐textured foreign body was visualized within the cervical canal, consistent with an ongoing infectious process (Figure 2). The cervical area was thoroughly irrigated with normal saline, after which the foreign material was carefully removed. The object had penetrated more than 5 mm into the cervical stroma, predominantly involving the anterior lip, and was excised as completely as possible. Subsequent inspection revealed multiple penetration sites measuring 1–5 mm across approximately four areas on the anterior lip and two on the posterior lip (Figure 3). Targeted biopsies were obtained from these sites for histopathological evaluation. Hysteroscopy examination proceeded thereafter, providing full visualization of the uterine cavity, which demonstrated no evidence of invasive pathology. All excised tissues were submitted for pathological analysis.

**Figure 4:**
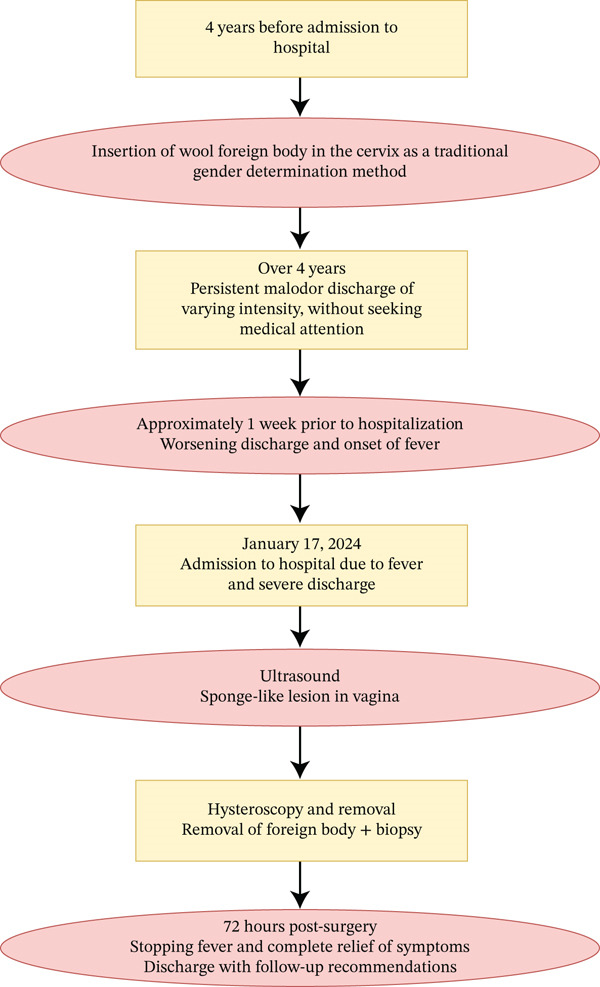
Timeline of clinical events.

Histopathological evaluation confirmed a foreign body reaction in the vaginal and exocervical tissues; characterized by both acute and chronic inflammatory infiltrates. The findings included reactive epithelial changes and clusters of keratin‐like fibers that resembled hair shafts infiltrating the cervical stroma, along with a fibrotic response. After the procedure, the patient was given a 72‐h course of empirical antibiotic therapy, which successfully resolved her fever and infection. She was discharged in stable condition without any complications and was instructed to follow a structured follow‐up schedule. This schedule consisted of two postoperative visits within the first month, followed by quarterly assessments over the next year with her treating physician. The clinical timeline is illustrated in Figure 4.

## 3. Discussion

The intentional insertion of a foreign object into the vagina is commonly observed in female children and adolescents; this behavior often stems from natural curiosity about their anatomy or may occur inadvertently due to vigorous perineal hygiene after urination. In some cases, it can also be associated with masturbation [2]. However, in our case, the patient was a middle‐aged woman, and the reason for the presence of the foreign object in her vagina was highly unusual. The purpose of this procedure was to achieve pregnancy while specifically ensuring the conception of a male fetus. Various objects can be intentionally inserted into the vagina for medical or personal reasons, including contraceptive devices, pessaries for treating uterine prolapse, medications for inducing abortion, and, unfortunately, items associated with sexual abuse [1, 3]. However, in this particular case, the patient had inserted a piece of wool‐like foreign body into her vagina, which is an extremely rare occurrence.

Priya Col et al. reported that tissue responses to retained spongiform material may present either acutely, characterized by abscess formation, or as a chronic fibrotic reaction that can manifest months to years after retention [4]. In the present case, the tissue response appears to have been chronic in nature, with a delayed acute presentation likely triggered by the gradual degradation of the wool‐derived material and subsequent inflammatory changes over 4 years.

Pushplata et al. reported three cases of vaginal foreign bodies, in which the most common clinical manifestations were foul‐smelling vaginal discharge and fever, typically presenting approximately 3 months after the introduction of the foreign object [1]. In contrast, the present case also involved foul‐smelling vaginal discharge accompanied by fever; however, these symptoms manifested 4 years after the foreign body had been introduced.

There are several diagnostic methods available for detecting vaginal foreign bodies: including pelvic radiography, magnetic resonance imaging (MRI), and ultrasonography. A systematic review by Umans et al. examined the detection of vaginal foreign bodies in pediatric patients; the review concluded that ultrasonography is the most appropriate and commonly used diagnostic tool, offering greater accuracy compared with radiography [5]. In the current case, both ultrasonography and CT were used to locate the foreign body, with the estimated dimensions closely matching the actual size of the object.

The prolonged retention of a foreign body in the vagina can lead to the formation of granulation tissue, changes in normal tissue, tissue adhesion, fibrosis, and more severe complications, such as vesicovaginal fistulas and even cancer. These issues often require invasive procedures, including complex and repeated surgeries [3, 6, 7]. In our case, the long‐term presence of a wool‐like object in the vagina resulted in ectocervical tissue showing both acute and chronic inflammation, along with reactive epithelial changes. Additionally, we observed clusters of keratin‐like fibers resembling hair shafts penetrating into the cervical stroma, accompanied by a fibrotic reaction.

The management of complications resulting from retained vaginal foreign bodies requires timely and accurate diagnosis, which can be facilitated through clinical examination and ultrasonography. Vaginoscopy remains the gold standard for both diagnostic assessment and therapeutic intervention [5]. In the present case, following initial clinical evaluation and ultrasonography, vaginoscopy and hysteroscopy were performed under general anesthesia. Upon removal of the foreign body, tissue specimens were obtained for histopathological analysis.

The primary limitation of this report is that, despite recommendations, the patient did not return for follow‐up after discharge. Nevertheless, the case demonstrates several strengths that enhance its scientific and clinical significance. It provides a comprehensive account of a rare clinical scenario involving the long‐term retention of a wool‐like vaginal foreign body for traditional medicine purposes. The diagnosis was confirmed through multiple modalities, including ultrasonography, hysteroscopy, and histopathological analysis. Moreover, the inclusion of high‐resolution imaging (Figures 1, 2 and 3) and a detailed procedural timeline (Figure 4) further contributes to the educational value of the report.

## 4. Conclusion

The retention of a foreign body within the vaginal vault may precipitate a spectrum of clinical manifestations and sequelae, contingent upon the nature of the foreign material and the protractedness of its persistence. For the discernment of such entities, an initial clinical examination, supplemented by ultrasonography interrogation, is recommended. The optimal therapeutic paradigm encompasses the performance of vaginoscopy and hysteroscopy. Should the foreign body have infiltrated contiguous normal tissue, attendant histopathologic alterations therein may be elucidated through biopsy. These findings, in turn, facilitate the formulation of tailored protocols for subsequent follow‐up evaluations.

## Funding

No funding was received for this manuscript.

## Consent

We extend our profound gratitude to the patient for her exemplary cooperation and participation in this investigation, with unequivocal assurances provided regarding the inviolate safeguarding of her privacy and personal particulars. Patient anonymity has been meticulously upheld throughout.

## Conflicts of Interest

The authors declare no conflicts of interest.

## Patient Perspective

The patient recounted her initial perception that the ethnomedical intervention was innocuous and efficacious in promoting male fetal sex selection. She had not anticipated the resultant morbidity or jeopardy to her overall health. Subsequent to therapeutic intervention and elucidation by the clinical team, she acknowledged the imperative of seeking professional medical counsel prior to engaging in any such practices. She advocates that individuals abstain from unverified traditional modalities absent corroborative medical guidance.

## Supporting information


**Supporting Information** Additional supporting information can be found online in the Supporting Information section. For additional details, the CARE Checklist has been provided as a supporting file.

## Data Availability

The pathology results, patient tests, sonography, and treatment progress reports are available from the first author upon request.
