# Supplementary material for: Unusual Case of Cervical Tissue Penetration by a Wool‐Like Foreign Body Inserted for Male Child Conception
Source: Case Rep Obstet Gynecol. 2026 Jun 7;2026:8965121. doi: 10.1155/crog/8965121 (PMC13243866; doi:10.1155/crog/8965121)
Supplement: Supplementary file 1 — Supporting Information Additional supporting information can be found online in the Supporting Information section. For additional details, the CARE Checklist has been provided as a supporting file. [file CROG-2026-8965121-s001.docx]

CARE Checklist of information to include when writing a case report

| Topic | Item | **Checklist item description** | Reported on Line |
| --- | --- | --- | --- |
| Title | 1 | The diagnosis or intervention of primary focus followed by the words "case report". | 1-2 |
| Key Words | 2 | 2 to 5 key words that identify diagnoses or interventions in this case report, including "case report" | 33 |
| Abstract(no references) | 3a | Introduction: What is unique about this case and what does it add to the scientific literature? | 18-19 |
|  | 3b | Main symptoms and/or important clinical findings | 18-19-20 |
|  | 3c | The main diagnoses, therapeutic interventions, and outcomes | 20-28 |
|  | 3d | Conclusion—What is the main “take-away” lesson(s) from this case? | 29-31 |
| Introduction | 4 | One or two paragraphs summarizing why this case is unique (may include references) | 38-39 |
| Patient Information | 5a | De-identified patient specific information | 44-45 |
|  | 5b | Primary concerns and symptoms of the patient | 45-47 |
|  | 5c | Medical, family, and psycho-social history including relevant genetic information | 45-47,53-56 |
|  | 5d | Relevant past interventions with outcomes | 45-47,53-56 |
| Clinical Findings | 6 | Describe significant physical examination (PE) and important clinical findings | 48-62 |
| Timeline | 7 | Historical and current information from this episode of care organized as a timeline | 145, Figure 4 |
| Diagnostic | 8a | Diagnostic testing (such as PE, laboratory testing, imaging, surveys) | 48-52, 57-62 |
| Assessment | 8b | Diagnostic challenges (such as access to testing, financial, or cultural) | 75-78 |
|  | 8c | Diagnosis (including other diagnoses considered) | 62-70 |
| Therapeutic  Intervention | 8d | Prognosis (such as staging in oncology) where applicable | 75-78 |
|  | 9a | Types of therapeutic intervention (such as pharmacologic, surgical, preventive, self-care) | 63-74 |
|  | 9b | Administration of therapeutic intervention (such as dosage, strength, duration) | 63-74 |
|  | 9c | Changes in therapeutic intervention (with rationale) | NIL |
| Follow-up and Outcomes | 10a | Clinician and patient-assessed outcomes (if available) | 78-83 |
|  | 10b | Important follow-up diagnostic and other test results | 78-80 |
|  | 10c | Intervention adherence and tolerability (How was this assessed?) | 78-83 |
|  | 10d | Adverse and unanticipated events | NIL |
| Discussion | 11a | A scientific discussion of the strengths AND limitations associated with this case report | 85-125 |
|  | 11b | Discussion of the relevant medical literature with references. | 85-125 |
|  | 11c | The scientific rationale for any conclusions (including assessment of possible causes) | 135-142 |
|  | 11d | The primary “take-away” lessons of this case report (without references) in a one paragraph conclusion | 135-142 |
| Patient Perspective | 12 | The patient should share their perspective in one to two paragraphs on the treatment(s) they received | 155-159 |
| Informed Consent | 13 | Did the patient give informed consent? Please provide if requested | YES |
